# Men’s participation in maternal and child health care in Western Uganda: perspectives from the community

**DOI:** 10.1186/s12889-019-7371-3

**Published:** 2019-08-05

**Authors:** Florence Muheirwe, Said Nuhu

**Affiliations:** 1 Valley University of Science and Technology, P. O. Box 44, Busenyi, Uganda; 2 Institute of Human Settlement Studies, Ardhi University, P. O. Box 35176, Dar es Salaam, Tanzania; 3 Swedish University of Agriculture Science, Uppsala, Sweden

**Keywords:** Participation, Maternal health care, Child health care, Uganda

## Abstract

**Background:**

Participation of men in Maternal and Child Health (MCH) is crucial for the reduction of infant and maternal mortality. Men may be influential in making health care decisions that may affect their female partner’s access to health care services, but also as individuals, whose health status has a significant impact on the health of their partners’ and that of their children. However, male involvement is still inadequate due to various reasons. This paper sought to explore the community perspectives towards participation of men in maternal and child health care in Kabale District, Western Uganda.

**Methods:**

The study used a case study approach. Household questionnaires, in-depth interviews, focus group discussions, direct field observation and document review were employed to collect data. One hundred and twenty-four respondents completed a household questionnaire, eight key informants took part in semi-structured interviews and thirty-six community members (two men and two women groups) participated in focus group discussions.

**Results:**

The participation of men in maternal and child health care was found to be low. Patriarchal community values and norms influencing gender roles hindered male involvement in MCH. More so, sensitisation on the importance of male involvement was inadequate.

**Conclusion:**

Men’s participation in MCH is affected by multiple factors emanating from the community and health institutions. Involving men in MCH is critical, and therefore participatory and comprehensive approaches should be applied to encourage participation. Sensitisation of communities is fundamental for increasing awareness of the significance of male involvement in MCH.

## Background

Maternal and Child Health care involves reproductive goals of: preventing unwanted pregnancies; decreasing high risk pregnancies; decreasing morbidity and mortality; and increasing accessibility to family planning services [1–3]. Due to its significance, Maternal and Child Health (MCH) is a key indicator for measuring development of nations [4]. Reducing infant and maternal mortality rates were incorporated in the Millennium Development Goals (MDGs) and are currently among the major yardsticks in the Sustainable Development Goals (SDGs) [5]. Reduction of maternal mortality (SDG 3.1) and infant mortality (SDG 3.2) is fundamental for ensuring a health life and promoting the well-being for all ages by 2030. Thus, MCH has been incorporated in development strategies of various countries in order to contribute to the global vision of 2030 [5, 6].

Globally, developing countries are leading with high maternal and infant mortality rates [7–9]. Sub Saharan Africa, is noted as having high infant mortality rates compared to other regions [8, 10]. There are also significant infant mortality rate variations within different regions in Sub Saharan Africa such as East Africa. Notably, Uganda is far much below the other East African Countries in reducing infant mortality [5, 6, 11]. In the 5th Uganda Demographic and Health Survey (UDHS) report of 2011, the number of mothers dying while giving birth in the country was 438 deaths per 100,000 live births, while infant mortality stood at 54 deaths per 1000 live births [12]. According to Ministry of Health in Uganda, the occurrence of maternal, neonatal, peri-natal and child deaths in the country, is a major concern for the government and all stakeholders [13]. Congruently, male involvement in MCH among other factors, can contribute to the reduction of maternal and infant mortality [6, 14].

Men are important in MCH care, not only as women’s partners, but also as individuals with significant reproductive impact on women’s and children’s health [14–16]. The involvement of men in MCH services facilitates the engagement with health service providers and therefore, presents an opportunity to acquire health education and access to health services for their own benefit [16]. This is crucial in preventing transmission of diseases’ to their partners, as well as the unborn children. Acquiring information enables men to make appropriate and effective health care decisions for their partners and children too [16–18]. This includes facilitating women to access and seek health facilities and utilisation of appropriate family planning methods [19]. Notably, male involvement in maternal health services also promotes awareness about obstetric danger signs [20], significantly improves communication among partners which leads to reduction of maternal workload during pregnancy and encourages postnatal care attendance [6, 18]. Consequently, infant feeding practices, childhood immunisation and care seeking for childhood illness is promoted [6, 18, 21]. Thus, male involvement improves maternal health utilisation reducing maternal mortality, as well as preventing HIV transmissions.

The participation of men in the broader perspective of safe motherhood is however still inadequate [14, 22, 23]. It is evident that in developing countries, husband involvement in maternal health care is particularly very low [24]. Infant delivery by skilled providers in Busia, Kenya was very low due to failure of men to support their partners’ to access health delivery services [25]. Studies conducted in Uganda, also indicate that male involvement in MCH is unsatisfactory. Mbale district, in Eastern Uganda, registered 74% low male involvement in Prevention of Mother to Child Transmission (PMCT) [26]. In Gulu, Northern Uganda, male involvement was registered as high during ante-natal visits, but it was noted that only men with Reproductive Health (RH) knowledge were willing to attend skilled Antenatal Care (ANC) [27]. A study conducted in Kabale, revealed that men were more active in maternal health care than in child health care however, their participation in the prevention of emergency obstetric referrals was inadequate [23]. Low participation of men in various societies is determined by varying and sometimes similar factors.

There is documented evidence on factors that influence men’s participation in MCH at individual, family, community and health facility levels [5, 28, 29]. It is noted that gender inequalities play a major role in maternal health [24, 30]. These inequalities are reflected in communities in regard to segregated gender roles, as well as at health facilities in the feminisation of programmes [6, 23, 24]. Although, traditionally men have not been involved in RH care of their partners, the shortcomings in the health care service delivery system such as poor attitudes of healthcare providers and ineffective programmes have equally discouraged their participation [6, 24, 31].

Despite the low involvement of men in MCH and barriers discouraging participation, studies have shown that men are involved in different ways. They participate in their children’s health care by either; taking children to hospital, accompanying their wives to take children, or meeting all required needs to improve their health status [28, 32]. In the Zulu community, Mullick et al. observes that a man, as a head of the family, is expected to enable his wife to meet her health needs [22]. This same sentiment is re-echoed by Kululanga et al. in Malawi [24]. The study conducted by Ntabona in Gambia reveals men’s concern as, covering the financial costs of ANC of their spouses amidst the polygamous syndrome [32]. In Uganda, Kakaire et al. notes that men were more active in maternal health care than in child health care, participating in various ways such as, making birth plans and meeting labour costs among others [28]. Contrary, a study by Muheirwe and Nuhu in Kabale District revealed that, men were more involved in child health because of their concern for children for prestigious reasons but not in maternal health [6].

The government of Uganda established strategies and policies focusing on reduction of maternal and infant mortalities and these include; the Health Sector Strategic Plan I 2009/2010 and Uganda National Population Policy for Social Transformation and Sustainable Development of 2008 among others [33, 34]. Despite this initiative, currently, the country’s maternal and infant mortality rates are at 336 deaths per 100,000 live births, and 22 deaths per 1000 live births respectively [34]. Although this has been applauded as a milestone from the previous alarming rates of 2011, a lot is still desired to ensure no woman loses life while giving birth and no child dies at infancy. It is agreed that low participation of men in MCH is one of the limiting factors to eradication of maternal and infant mortality [6, 18].

Studies have been conducted in Uganda regarding male participation. However, these focused on specific aspects of maternal health that affect men as well such as PMCT and RH [26, 27]. These studies were also conducted in different regions. Nonetheless, some of the factors cannot be generalised since regions vary in economic and infrastructure development, literacy levels and culture as well. The study by Muheirwe and Nuhu focused on institutional factors determining male participation in MCH in Kabale District [6]. Factors contributing to low participation of men in MCH particularly from the community perspective have not been adequately explored. Therefore, this paper explores the community perspectives towards men’s participation in MCH in Kabale district, Western Uganda. The district is one of the patriarchal societies in Uganda dominated by cultural values and norms that influence gender roles in the communities. This is to assess whether these cultural values and norms have hindered men’s participation in MCH in Western Uganda. The information will be vital for designing interventions that will contribute to the reduction of maternal and infant mortality in the country and thus, contributing to achieving the SDGs.

## Methods

### Study design and area

A case study approach was used because male involvement in MCH is a complex issue, but also a contemporary phenomenon. It is determined by multiple factors and has dominated global and national debates. The approach enabled exploration of the communities’ views towards male involvement in MCH which is determined by various issues from the community, as well as at health facilities. It also facilitated the selection of the study area and enabled the application of multiple data collection techniques. Kabale district, in Western Uganda was chosen purposively as the study area because it is one of the patriarchal societies, highly populated but with the lowest hospital deliveries [6, 35]. The district also belongs to Kigezi region with hospital deliveries at 69.7%, which is the second lowest in the whole country [34]. Infant mortality is at 45%, access to ANC services is 56.1% out of 181 births accessed, and accessed postnatal care is 69.7% out of 507 births [34]. These statistics refer to access in terms of hospital deliveries according to the UDHS survey. The district infant mortality is the third highest in the country [12, 23]. Rubanda and Rukiga health centers were purposively chosen because they serve the ‘hard to reach and hard to serve’ areas. Local leaders at Rubanda and Rukiga sub counties availed the list of parishes under their jurisdiction. Parishes in which the study was conducted were randomly selected and these included Nyakabungo, Nyarurambi (Rubanda), Mparo and Noozi (Rukiga).

### Data collection methods

In collection of data, the methods used included: household questionnaires, in-depth interviews, Focus Group Discussions (FGDs), direct field observation and document review. Household questionnaires’ enabled the collection of demographic data in order to understand the gender perspectives, awareness of MCH, as well as perceptions of the community towards men involvement in MCH. The researcher(s) administered the questionnaires from the households at the respondents’ convenient time. The questionnaires included both closed and open-ended questions. The latter enabled respondents to express freely and discuss at length the themes asked, while the former, guided the flow of the discussion on specific topics of interest. Interviews allowed participants to express themselves deeply without hindrances, as the researcher took note of their opinions as well as expressions. FGDs facilitated the collection of information in groups to gather gender segregated views and opinions from participants regarding male involvement in MCH, its significance and barriers to male participation. These were single-sex groups to enable free interaction and voicing of opinions among peers. Participants in the FGDs were from different villages to allow freedom of expression and security of privacy. Participants did not want to be recorded but they allowed the researcher to take notes during the discussion and interviews. Direct field observation was used to view gender participation and communication materials at health facilities. The administrators of the health centers were briefed about application of this technique. The respondents were de-briefed during FGDs. Through document analysis, various published and unpublished literature was reviewed which included among others, MDGs, SDGs, Uganda Health Sector Strategic Plan II, Uganda National Population Policy for Social Transformation and Sustainable Development, Uganda Health Demographic Surveys and health records from the district and at health centers. This enabled collection of health demographic information, data on the state of male participation in MCH, and contributed to understanding health strategies and programmes in the country and at a global level.

### Study participants

Purposive and random sampling was used to select households and key informants. Respondents (*n* = 124) with children and living with partner(s) were selected. The selection of households was based on the list obtained from local leaders. The head of the household (male or female), was selected. Thus, one hundred and twenty four respondents came from 124 households. Key informants (*n* = 8) who included two Traditional Birth Attendants (TBAs) and two Village Health Team (VHTs) members were purposively selected because of their role in the health care of community members. TBAs are instrumental in communities where the services of midwives cannot be easily accessed. They assist in emergence birth deliveries in communities for women who either, may not access hospital delivery services due to financial, health facility accessibility constraints or others. VHTs are selected by local authorities with the aid of health officials. They assist the health officers in offering health services in the community which include first aid, monitoring patients and mobilising communities for participation in health programmes. One health official from the district in charge of MCH, two heads of departments for maternal and pediatric wards and one official in charge of the health center were also purposively selected. A total of 36 respondents participated in the FDGs. In each health centre, 2 FGDs (one for women and another for the men group) were held. Fifteen participants in Rubanda health centre and 21 from Rukiga Health Centre IV were involved in FGDs. Out of 15 participants in Rubanda, 8 (53%) were female and 7 (47%) were male, while out of 21 participants in Rukiga, 12 (57%) were female and 9 (43%) were male. Participants in these discussions included community members that were not subjected to the questionnaire or interviews. They only participated in the FGDs to avoid recycling of same views from same people but in different approaches.

### Data analysis

Quantitative data was analysed using the Statistical Package for Social Sciences (SPSS) and was presented as descriptive statistics (see Tables 1, 2 and 3). The information gathered covered age, sex, education and marital status. It also included reasons for male participation and its significance. Content analysis of information from qualitative data was done in accordance to emerging themes as presented in the result and discussion sections. The study did not use audio visual gadgets to collect data therefore, the information given in the quotations in this paper has been paraphrased paying attention to maintain the original meaning.

**Table 1:** Respondents characteristics (*N* = 124)

Age	Sub-health district				Education		Marital status	
	Rubanda		Rukiga					
	M	F	M	F				
18–28	14	19	9	21	Not educated	14	Religious marriage	37
29–39	6	8	6	6	Primary education	73	Traditionally marriage	55
40–50	8	2	16	4	Secondary education	26	Cohabitating	32
51+	2	3	0	0	Diploma/College	10	Total	124
Total	30	32	31	31	Degree	1

**Table 2:** Reasons why men do not participate in MCH (*N* = 124)

Reasons for not Participating	Sex			
	Male		Female	
	Number of Responses	%	Number of Responses	%
Lack of interest	42	68	31	49
Men not required to participate	3	5	1	2
Facility not designed for men	1	2	4	6
Men always busy	12	20	19	30
Inaccessibility of facilities	1	2	2	3
Men always far away from home	2	3	6	10
Total	61	100	63	100

**Table 3:** Reasons for importance of male involvement in MCH (*N* = 48)

Reason for importance of male involvement	Number of Respondents	%
Getting couple VCT services	23	48
Offering support, love and care	15	31
Encourages faithfulness and trust	7	15
Gaining knowledge on MCH	3	6
Total	48	100

## Results

### MCH awareness and education

Awareness and education are fundamental components for changing attitudes in a community. It was thus vital to explore community awareness of MCH and male participation. During the discussions, it was revealed that communities were aware of the campaigns for male involvement in MCH by the government, but had not received adequate health education about its implication. Announcements had been made in public gatherings inviting men to actively participate in pre-natal and postnatal care. A few men had responded positively to this call, while the majority had ignored it as another ‘government useless program’, as one male respondent commented:

“There was an announcement in church for a whole month that women going for ante-natal care are encouraged to go with their husbands, but there was no explanation given why this was important.”

This was also confirmed by one of the health officials saying:

“We have been announcing in public places that all pregnant women should come along with their partners, but people do not take respond to these announcements.”

After probing, the official explained that they had been conducting awareness campaigns at health facilities only because of insufficient funds, to enable conducting mass mobilisation and education programmes in the communities. In one of the FGDs, men lamented that VHTs in communities were not helpful in educating communities as well. During the interview with one member of the VHTs, it was revealed that the VHT committees are not facilitated and therefore were incapable in helping community members.

FGDs with women also revealed that health officials encouraged antenatal attendance of couples with inadequate justification. It was obvious only when a woman had health complications, that an official invitation to the partner would be sent as one respondent confirmed:

“If you see a health worker giving you a letter inviting your husband, just know that there is a big problem. If you are not the one who has reported your husband for not buying you clothes or good food, then you sense that either your child, your-self or both of you are at a health risk”

Revelations from the TBAs also portrayed that men rushed for their services when their partners experienced emergency labour pains. This is an indication that male involvement is mandatory in emergency conditions that demand attention from both partners, for appropriate health decision making in the family. It was also revealed that the district had embarked on a feasibility study on male involvement that was never accomplished due to inadequate facilitation, and therefore, implementation of health education remains lacking.

The findings from discussions with men and women were in harmony that male involvement was vital, if only the men could understand their role and its health implication for both mother and child, but also for appropriate decision making. It was also agreed that the community lacked adequate health education that is vital for changing attitudes towards men involvement in MCH.

### Social cultural values and MCH

It was vital to find out whether the respondents ranked male involvement as an important aspect in MCH and ascertain specific attitudes and perceptions. Out of 124 respondents, 39% (*n* = 48) confirmed that male involvement was important, 55% (*n* = 68) thought it was not, and 6% (*n* = 8) were indifferent. However, the respondents who thought that male participation in MCH was not important said that, MCH was a women’s issue and thus men were not directly impacted. Most respondents concurred that men do not conceive nor do they give birth and for this reason, they had no business at health facilities and specifically in MCH programmes. In the male group discussions, one respondent remarked:

“…why should a man go to hospital and yet he is not pregnant? These things that you people of the young generation are demanding, like being accompanied to a health facility, are less important. Let issues that involve men be for men and those involving women be for women…”

This confirms that gender stereotyping hinders male involvement in MCH and cultural values were still inherently significant in the area. It was observed and also stipulated by respondents that sister’ in-laws and mothers’ in-laws were always cooperative in MCH. In the discussion with the women, there was consensus that in-laws always stood up for them in their times of MCH needs. One respondent commented:

“My husband does not have to be with me, since my mother in-law is always accompanying me; instead, he has to go and work in order to get food that will feed us and provide other hospital requirements’”.

Communities in this area still live under communal settings whereby relatives live within the same area, otherwise it would be impossible to rely on in-laws living a greater distance away.

During FGDs for both women and men, it was revealed that the attitude towards male involvement was negative when a man was identified as often accompanying his wife for MCH services. The discussions revealed that the community thought that the couple was either publically displaying their love, did not trust each other, or the woman had bewitched her husband. In the FGDs with the men, one of the respondents commented:

“In my village, my own mother told me that my wife is a witch because I am always following her to the health center. My male friends scorned me, but for me, I do not care because I love my wife and I want to always be there whenever she needs me”.

The fact that male involvement in MCH could be attributed to witchcraft or public display of affection, not only shows the traditional values and culture in the study area, but also implies that the significance of male involvement is far from being understood by such communities. It was important to stipulate more reasons that limited the participation of men in MCH as indicated in Table 2.

Findings in Table 2 reveal that, 68% (*n* = 42) out of 61 male respondents and 49% (*n* = 31) out of 63 female respondents mentioned lack of interest as the main reason for limited participation of men in MCH. During the discussion in focused groups, it was pointed out that men lacked interest simply because they have not been sensitised about the significance of their participation in MCH as earlier noted.

Both female and male respondents attributed low participation of men in MCH to male gender roles. As findings in Table 2 indicate, 30% (*n* = 19) out of 63 female respondents and 20% (*n* = 12) out of 61 male respondents argued that, most men were occupied with meeting the financial needs of their families and engaging in agricultural activities. The justification that men are always busy has a social cultural implication emanating from the division of labour in regard to social roles. Discussions with participants from the health center revealed that challenges of male involvement in MCH include the fact *‘that some men in the area leave a careless life of drinking in bars’* disputing the claim that men were always busy.

The reason that facilities were not designed for men, though it did not come out as one of the reasons with the highest score, cannot be undermined. This shows the feminisation of services emanating from the social cultural aspects of the difference between men and women in the community. It was argued that men are not required to attend MCH programmes because they are implemented by female nurses. Communication materials in the hospital in form of posters portraying women roles in maternal responsibilities were also observed. Some of the men who had accompanied their partners’ were observed in the compound waiting for the health workers to talk to them alone. When asked, one of the men who was waiting for his partner being attended to in the health facility commented:

“This is a maternity ward for women, what will I be doing there as a man since men do not get pregnant? Even this facility is full of female nurses; I cannot tell them my health problems”.

This kind of attitude towards health facilities is influenced by social and cultural values. One of the health workers testified that indeed, involving male participation was a challenge because men are not willing to participate and summed it up that:

“Men are not willing to escort mothers because pregnancy is not illness…thus find no need to go with their partner for MCH services…”

The fact that 10% (*n* = 6) of the female respondents and 3% (*n* = 2) of the male respondents argued that men were always far away from home and therefore, were not able to get involved in MCH was also crucial. It should be noted that in the study area, both health centers’ are located in one of the hard to reach and hard to serve populations. Men from these communities leave their homes in search of greener pastures to earn income and support their families. In Rubanda Sub County, men go to the mines or forests for logging, while those from Mparo, go to the city or big towns looking for employment opportunities. Health workers also concurred that men go far way because of financial obligations that normally rests heavily on men culturally. In the words of one of the health officials:

“Men in this area go to work in cities to get money for taking care of their families’ needs…they come back to impregnate their wives and go back especially men who are still young and energetic…Even the women encourage them to go away”.

During FGDs however, it was noted that most of the middle aged male respondents were of the view that male involvement in MCH was a new phenomenon. They observed that in the previous decades, health officials did not care whether you were accompanied by your husband or not. The findings from this study confirm that social cultural factors hinder men’s participation in MCH, and that these factors cannot be alienated from socioeconomic factors.

### Significance of male participation in MCH

Despite the low participation of men in MCH influenced by limited awareness and social cultural values, the study sought to explore whether communities could stipulate the significance of male participation. Forty eight (39%), out of 124 respondents, who thought male involvement was important as presented in the previous subsection, were asked to justify their reasons. Table 3 sums up their views.

The findings in Table 3 show that male involvement in MCH was important because of the services couples receive such as Voluntary Counseling and Testing (VCT). This was attributed to the integration of PMCT and Elimination of Mother to Child Transmissions (EMCT) programmes, where couple counseling for VCT is inevitable as revealed during the interview with one health official:

“PMCT and EMCT programmes are MCH care services, in which VCT is a compulsory component, for the effective implementation of the programmes and some men have started to realise that their participation is crucial, not only for their health status, but that of their partners and children as well”

Respondents who thought love, care and support were the main reason for the importance of male involvement in MCH noted that, when a husband accompanied his wife at the health center, he would be there to support her in case of any health failings, or provide financial support required. This is noted from the comment of one of the respondents:

“…when I come along with my husband for ante-natal, the health workers can tell him to buy for me a maternity dress and nutritional foods for an expecting mother and he subdues… but when I come alone and tell him these requirements’, he thinks I just want good things and I am using the pregnancy as an excuse…”

Male involvement is important as portrayed because it influences men to care and support their wives in a number of ways and this support is critical as well for MCH. This was also re-echoed by one of the officials from the district saying:

“Men are important in MCH because they are the ones that may give permission to a mother to utilise any MCH services, but also provide support such as transport and other requirements”.

Health care decisions are predominantly taken by men on behalf of their families. Notably, respondents who attributed the importance of male involvement in MCH to gaining knowledge in MCH were few.

Women group discussions revealed that when a woman is escorted by her partner for either antenatal or postnatal care, the health officials attended to the couple first, and hence were able to leave the health facility before others. One of the health officials confirmed that this is done to encourage spousal counselling and male participation in health care of their families.

Perspectives from the community regarding male participation in MCH revolved on education and awareness as well as social cultural factors. The significance of male participation was also acknowledged, if sensitisation programmes are rolled out in communities.

## Discussion

The role of men in MCH was brought to policy makers during the Cairo International Conference on Population and Development (ICPD) Programme of Action in 1994 [8, 36]. The conference recognised that men have shared responsibility and active involvement in responsible parenthood, sexual and reproductive behavior. The role of men in transforming the social roles that constrain RH and rights was also acknowledged [8, 37]. Men were recognised because of their impact on women’s and children’s health as decision makers and thus agents of change. It is noted that involving men in family planning and RH, pre and postnatal care as well as child care improves parenting and prevents the spread of Sexually Transmitted Infections (STIs) and Human Immunodeficiency Virus (HIV), which improves maternal as well as child health [14, 19, 38, 39]. Unfortunately, men rarely accompany their wives for antenatal clinic and medical check-up, nor do they take a leading role in their children’s health [14, 18, 40]. The reluctance of men’s participation is attributed to limited awareness due to inadequate sensitisation and health education programmes in communities.

Limited awareness regarding the significant contribution of male participation towards reducing infant and maternal mortality is still evident in most developing countries especially in rural communities. In Africa, limited awareness on the role of men’s participation in MCH is looming [9]. In Western Kenya for example, lack of awareness was highlighted as contributing to low men involvement in Sexual and Reproductive Health [41]. In Tanzania, inadequate awareness contributed to low participation of men in PMCT [42]. Lack of awareness and sensitisation in the Zulu community in South Africa was noted as a barrier to men’s participation in RH too [22]. In Gambia, men were ignorant about the importance of completing the recommended four ANC visits [43]. Inadequate government initiatives in raising awareness in communities on the importance of male participation in health programmes due to resource constraints could be the stumbling block in most of the poor countries. However, low participation of men in health programmes cannot only be aligned to limited awareness only. In Uganda, economic factors such as lack of financial resources to meet financial costs for health care services and transport means to access health facilities are barriers to male involvement in health care programmes [18]. Notably socially constructed gender roles and health-system-related barriers such as poor attitudes of health workers, feminisation of programmes have also been highlighted as significant barriers to male participation in health services in Uganda and Tanzania (6, 17]. Limited awareness is partly fueled by social and cultural values.

Social cultural values have been vehemently noted as discouraging male participation in health programmes. It is uncommon for men in African communities to take their children to hospital unless the mother is unwell [6, 14, 22]. Most men also do not accompany their partners to family planning, or ANC consultations and during labor, or delivery. It is documented that men in Kenya, Uganda, and South Africa are not involved in pre-natal and postnatal activities because clinic issues are culturally perceived as womens’ affairs [9, 22, 24, 25]. Child birth is considered a shameful process for husbands to witness and peer influence plays a role in discouraging supportive partners. Notably, social values determine gender roles which designate duties associated with men and women. In most counties, men provide resources to meet the health needs of women and their children [23, 24]. It should be noted that lack of interest by men in MCH services may be built through social values and cultures determining social roles and thus influencing or discouraging participation. However, financial factors cannot be isolated from social cultural values since social and cultural values have been revolving over the years along economic trends.

This study explores a new dimension of considering opinions from the grass root and from community members of various designations, to understand holistically barriers to male involvement in MCH. Several studies have looked at different dimensions on this topic. Scholars have focused on male participation in RH especially family planning and VCT [31, 41, 42, 44]. Participation of men in such programmes is relatively high because they benefit directly as health consumers, and therefore, the accrued health benefits for their partners and unborn children may be secondary. Some studies have concentrated on the gender dynamics as barriers to men participation in health programmes [4, 18, 29, 30]. Notably, gender roles are neither static nor universal. They are culturally constructed and vary from one society to another, therefore, this information overtime can be irrelevant when the culture and values are transformed. Thus, new information from the communities to update the literature periodically is paramount. Other studies have reviewed interventions for male involvement in health programmes [4, 6, 14, 17, 21]. These studies focus on institutional factors and target policy makers and practitioners. Community people for whom these interventions are designed have not been central in these studies. Therefore, this study focuses on a broader perspective of women and children’s health congruently, because maternal health has implications for children’s health. It also explores opinions on men participation in MCH from various stakeholders in the community, health facilities and departments.

Findings from this study have implications for policy makers, practitioners and other stakeholders. Development partners and Civil Society Organisations (CSOs) are fundamental in supplementing and enabling governments’ efforts in terms of resources, and sometimes in implementation of program activities. Integration and collaborative mechanisms are therefore essential to enable relevant ministries, departments and stakeholders to work towards encouraging male participation in health programmes in general. This ultimately could contribute to achieving the national health goals as well as SDG 3 targets 1 and 2.

However, this study was not without limitations. Only two health centers from the rural area in the District were considered for the study and therefore, the findings may not be accurately generalised to urban or peri-urban areas within the district, or other regions within the country, or other societies in other countries. Notably, it was not possible to access couples together, although this would have given a more authentic view on the study. This explains the variations in the number of respondents between male and female as well as the views. Undoubtedly, different categories of people in society have different opinions and views towards male participation in MCH. It would be significant to explore the perspectives of women towards ways in which men participate in MCH. This would reveal whether the ‘type’ of participation is satisfactory or not.

## Conclusion

Determinants for men’s participation in MCH range from individual, community and institutional factors. Perspectives from the community indicate that, social cultural values have been discouraging male participation significantly in MCH compared to other factors. However, the significance of male participation in contributing towards better health outcomes for women and children should not be undermined. Therefore, addressing low participation of men in MCH, should focus on comprehensive and participatory measures. Appropriate MCH programmes for men at health facilities, community education and health sensitisation campaigns to change the prevailing social-cultural attitudes and perceptions is vital. Making use of the established systems such as VHTs and other community social gatherings would be helpful in demystifying misconceptions, changing perceptions and attitude, consequently encouraging male participation over time.

## Data Availability

The data analysed during the current study are not publicly available because it is still going to be used but are available from the corresponding author on reasonable request.

## Abbreviations

ANC: Antenatal Care
CSOs: Civil Societies Organisations
EMCT: Elimination of Mother to Child Transmissions
FGDs: Focus Group Discussions
HIV: Human Immunodeficiency Virus
ICPD: International Conference on Population and Development
MCH: Maternal and Child Health
MDGs: Millennium Development Goals
PMCT: Prevention of Mother to Child Transmission
PRB: Population Reference Bureau
RH: Reproductive Health
SDGs: Sustainable Development Goals
SPSS: Statistical Package for Social Sciences
STIs: Sexually Transmitted Infections
UDHS: Uganda Demographic and Health Survey
VCT: Voluntary Counseling and Testing
VHTs: Village Health Team
